# Influence of Posterior Cruciate Ligament Resection on Knee Balancing and Bone Resection Thickness in Patients Undergoing Robotic-Assisted Total Knee Arthroplasty: A Prospective Study

**DOI:** 10.7759/cureus.78215

**Published:** 2025-01-29

**Authors:** A.B. Suhas Masilamani, Tarun Jayakumar, Praharsha Mulpur, Rakesh Patil, Kushal Hippalgaonkar, A.V. Gurava Reddy

**Affiliations:** 1 Orthopaedics, Institute of Orthopaedics and Robotic Surgery, Renova Century Hospital, Hyderabad, IND; 2 Orthopaedics, Sunshine Bone and Joint Institute, KIMS-Sunshine Hospitals, Hyderabad, IND

**Keywords:** pcl retaining, pcl sacrifice, posterior cruciate ligament, robot-assisted tka, total knee arthroplasty

## Abstract

Introduction

The decision to retain or sacrifice the posterior cruciate ligament (PCL) during total knee arthroplasty (TKA) is debated among surgeons. This study aimed to determine the effects of PCL removal on gap balancing, bone-cut thickness, and component positioning.

Methods

This prospective study included 70 consecutive patients with varus deformity undergoing Mako (Stryker Orthopaedics, Fort Lauderdale, FL, USA) robot-assisted TKA between January 2022 and June 2022. Flexion and extension gaps were initially captured with the PCL intact, using the mechanical alignment start-point, after which the knees were balanced using the functional alignment philosophy. Following this, the PCL was sacrificed, and gaps were recaptured to assess the dynamic change in balance and bone cut thickness, with statistical analyses performed.

Results

The mean age of the population was 59.46 years (SD = 9.2), with a female preponderance (N = 50, or 71.40%). PCL resection significantly increased the mean flexion gap in the medial compartment from 15.7 mm (SD = 2.36) to 18.8 mm (SD = 1.83), and in the lateral compartment from 22.7 mm (SD = 1.66) to 24.5 mm (SD = 1.62), with minimal change in the extension gap. Rebalancing after PCL resection resulted in a reduction in posterior femoral bone resection thickness, with the posteromedial cut decreasing from 9.6 mm to 7.1 mm, and the posterolateral cut from 4.8 mm to 2.7 mm.

Conclusion

This study demonstrates that PCL resection consistently leads to a preferential increase in the flexion gap, with minimal impact on the extension gap. The flexion gap increased more significantly in the medial compartment than in the lateral compartment, in approximately a 3:2 proportion. Additionally, PCL resection was associated with decreased posterior femoral bone resection thickness and a trend toward reduced femoral component external rotation.

## Introduction

Surgeons' preference for posterior cruciate ligament (PCL) retention versus sacrifice has been a long-standing debate in primary total knee arthroplasty (TKA) [1,2]. The soft tissue envelope around the knee is vital for stability and for restoring native knee kinematics [3,4]. The medial and lateral collateral ligaments provide varus and valgus stability in the coronal plane, while the PCL serves as the primary restraint for posterior tibial translation during flexion and for coronal plane stabilization in full extension [5].

Previous studies indicate that patient-reported outcomes in TKA are comparable whether the PCL is retained or sacrificed, with cruciate-retaining (CR) and posterior-stabilized (PS) implants used, respectively [6-8]. However, achieving the correct PCL tension during surgery is crucial, as it affects the tibio-femoral contact position, polyethylene insert pressures, and stresses at the implant-bone interface. An overly tight PCL can cause pain, restricted range of motion (ROM), and increased medial polyethylene loading, whereas a loose PCL can lead to flexion instability and implant failure [9].

The impact of PCL resection on flexion and extension gaps in TKA has been studied, with most studies reporting a preferential increase in the flexion gap with PCL resection or sacrifice [10-15]. However, the majority of these studies were conducted in vitro on cadaver specimens or in vivo with mechanical soft tissue tensioners, potentially lacking the elasticity of living tissues.

Kayani et al. first investigated the effect of PCL resection on flexion-extension gaps during robotic-assisted TKA (RA-TKA) [16]. They reported that PCL resection affected the flexion gap more than the extension gap, resulting in medio-lateral laxity in flexion. However, this study did not examine the impact of PCL resection on bone-cut thickness, femoral implant rotation, or sizing. Understanding the impact of CR and sacrifice on femoral component rotation, as well as the bone-resection thickness needed to balance the knee, is essential. Additionally, it is possible to compare these parameters before and after PCL retention by using dynamic ligament tension assessment with Mako software (Stryker Orthopaedics, Fort Lauderdale, FL, USA).

The primary aim of the study was to evaluate the influence of PCL retention and sacrifice on flexion-extension gaps, bone cut thickness, and component positioning in end-stage osteoarthritis (OA) knees undergoing RA-TKA. We hypothesize that PCL sacrifice is more bone-conserving, with reduced bone resection thickness, compared to CR-TKA.

## Materials and methods

This study was a prospective study of patients undergoing primary RA-TKA at a single high-volume arthroplasty center between January 2022 and June 2022. The robotic system used was the Stryker Mako System. The study was approved by the Institutional Ethics Committee (SIEC/2022/489) and was performed in accordance with the guidelines of the Declaration of Helsinki [17]. Patient records were prospectively collected in the Institutional Joint Registry database to record demographic data, surgical variables, and peri-operative complications, which were accessible only to all the authors.

Patients with primary end-stage OA of the knee associated with varus deformity were eligible for participation in this study. The study included 100 consecutive RA-TKA cases, performed by a single surgeon during the study period. Inclusion criteria consisted of patients with primary unilateral end-stage OA of the knee with varus deformity less than 20 degrees and flexion contracture less than 20 degrees. Patients with valgus deformity of the knee, prior high tibial osteotomy (HTO), post-traumatic and inflammatory arthropathy, coronal or sagittal deformity greater than 20 degrees, a previous history of knee surgeries, and BMI greater than 40 kg/m² were excluded.

Surgical technique

All patients were operated on through a midline incision and a medial parapatellar approach. Initial medial soft tissue release was performed until the deep medial collateral ligament (MCL). The anterior cruciate ligament (ACL) was removed if present, and the PCL was initially retained in all cases before soft tissue balancing commenced. After joint exposure, bone pins for the optical arrays were placed in the tibia and femur through extra-articular stab incisions. Medial femoral and tibial osteophytes were removed with a rongeur after the completion of landmark and image-based bone registration. All cases were operated on with a cemented Stryker Triathlon PS prosthesis (Stryker, Kalamazoo, MI, USA).

Pre-operative component positioning and alignment

The pre-surgical plan, or starting point for soft tissue balancing, was set to mechanical alignment with 0 degrees varus/valgus for both femur and tibia components. All cases were balanced based on the philosophy of functional alignment. The tibial slope was set to 3 degrees in all cases. Overall limb varus (hip-knee-ankle, or HKA) up to 5 degrees was accepted, with femoral alignment of 0-2 degrees of varus and tibial component alignment of 0-3 degrees of varus. Femoral component external rotation of 0-5 degrees was accepted. The tibial slope was fixed at 3 degrees to understand the influence of the PCL on the flexion gap in isolation from other variables.

Soft tissue balancing strategy

After osteophyte removal, joint spacer “spoons” or osteotomes are employed to tension the collateral ligaments in flexion and extension, and the gaps are recorded [18]. Soft-tissue joint balancing (dynamic balancing) is based on the quantitative representation of soft-tissue tension in extension and flexion. “Pre-balance,” as described by Lustig et al., is the ability to achieve medio-lateral flexion and extension balance “pre-emptively” and virtually, only by changes in component positioning in the coronal, sagittal, and axial planes, before bone resection, to avoid or minimize soft-tissue releases [19].

Stage 1

**Figure 1:**
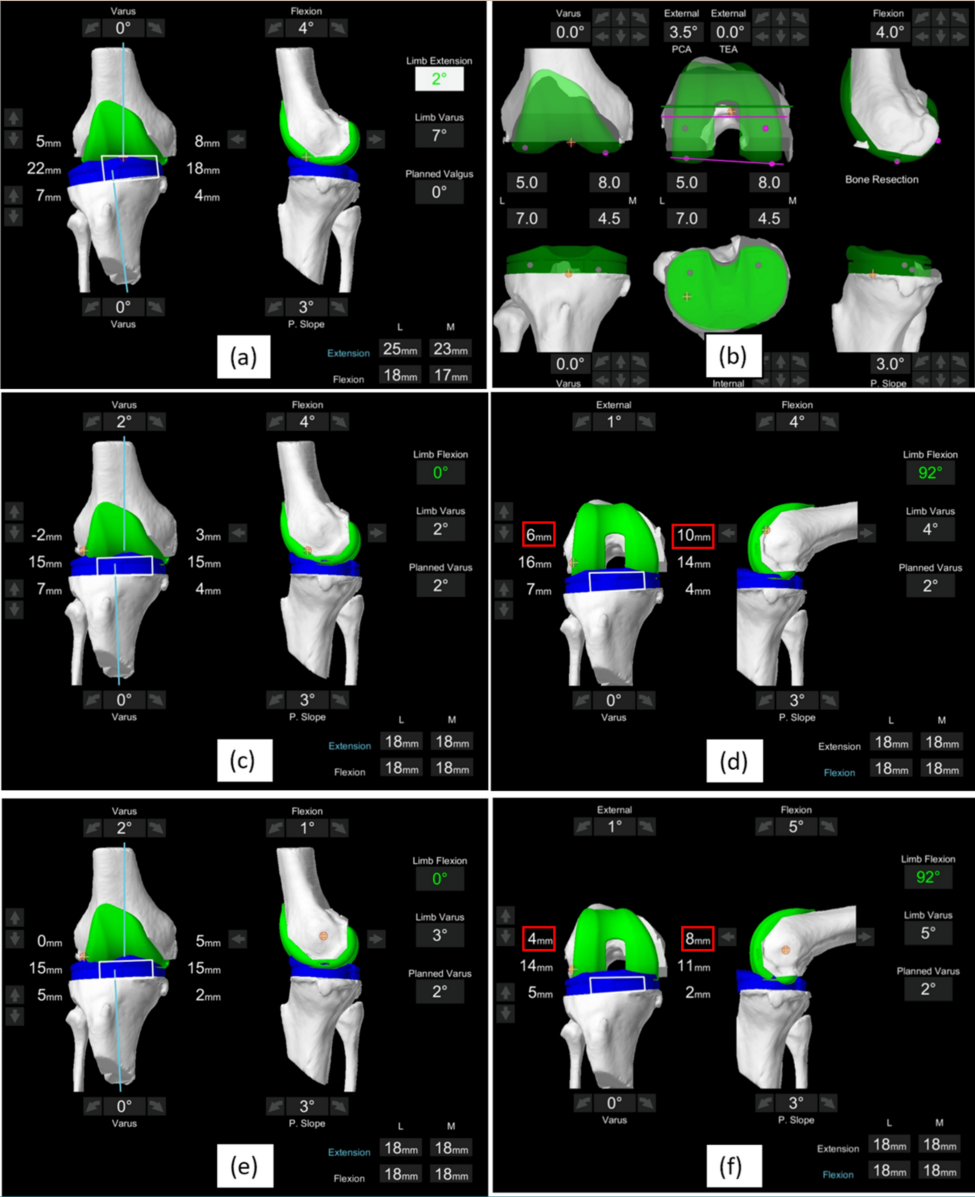
A MAKO pre-plan software image (a) Initial deformity and gap capture values; (b) Initial implant positioning set to mechanical alignment, with a fixed 3-degree posterior slope; (c)-(d) Extension and flexion (red box) bone cuts and gaps of the knee balanced with PCL retention; (e)-(f) Extension and flexion (red box) bone cuts and gaps of the knee balanced after PCL sacrifice (note the reduction in bone cut thickness in flexion, after PCL resection). This image was created using MAKO (Stryker Orthopaedics, Fort Lauderdale, FL, USA) pre-plan software.

In this case series, all knees were balanced based on the principles of functional alignment, with the PCL preserved in all cases to start with (Figures 1a-1d). A knee was defined as “well-balanced” or “pre-balanced” if the target 18 mm gaps were achieved, with a tolerance of lateral laxity (1 mm in extension and 2 mm in flexion). Based on this definition of balance, knees were classified as balanced or unbalanced with the PCL intact. The balanced gap values, bone resection thickness (in mm), and femoral component rotation were documented at this stage, but no bone resections were performed.

Stage 2

The PCL was subsequently removed in all cases; the flexion and extension gaps were recaptured. Based on the changes in the gaps, the knees were again balanced using the functional alignment philosophy with the same boundaries. New gap captures (before and after balancing), bone resection thickness (in mm), and femoral component rotation were again measured to determine the impact of PCL sacrifice on these parameters (Figures 1e-1f).

Variables assessed include dynamic gap capture values with the PCL retained, comparison of bone resection thickness, coronal limb alignment, and femoral component rotation between the two groups.

Statistical analysis

A sample size of 70 patients was determined to ensure a statistical power of 95% (1-β) for evaluating alterations in medial and lateral gaps during both extension and flexion phases. This calculation was based on an effect size of 0.30 and an alpha level of 0.05 [12]. The normality of data was assessed using the Shapiro-Wilk test. Baseline characteristics were compared between both groups. Categorical variables were evaluated using the Chi-square test, and the independent samples t-test was used to analyze continuous variables. Normally distributed data were evaluated using the independent samples t-test, and non-parametric data were evaluated with the Mann-Whitney U-test. All results are presented as mean (with standard deviations) and 95% confidence intervals. A p-value less than 0.05 was considered statistically significant.

## Results

Tables 1-2 show the baseline demographic characteristics of the population. The mean age was 59.4 (SD = 9.2), with the majority of females (N = 50, or 71.40%). The mean pre-operative coronal plane deformity of the knees in the study was 10.5 (SD = 1.9), and the majority of the patients belonged to the American Society of Anesthesiologists (ASA) grade 2 (N = 49, or 70%), with a mean Charlson Comorbidity Index (CCI) of 2 (SD = 1.2).

**Table 1:** Baseline demographics of the study population

Parameter	Mean	SD
Age (years)	59.46	9.2
BMI (kg/m^2^)	29	4.19
Charlson comorbidity index (CCI)	2	1.2
Pre-operative coronal deformity (degrees)	10.5	1.9
Tourniquet time (minutes)	57.7	11.1

**Table 2:** Gender and ASA grade distribution of the study population ASA, American Society of Anesthesiologists

Parameter	Number	Percentage
Gender distribution; female	50	71.40%
ASA grade distribution		
1	17	24.30%
2	49	70%
3	4	5.70%

All 70 knees were balanced virtually with Mako dynamic joint balance assessment initially, while retaining the PCL. Initial flexion-extension gap capture values, with and without the PCL, are shown in Table 2. Sacrificing the PCL was shown to mainly influence and increase the flexion gap of both the medial and lateral compartments, which was statistically significant. There was a minimal change in the extension gap values, with or without the integrity of the PCL. The mean lateral flexion gap increased from 22.7 mm (SD = 1.66) to 24.5 mm (SD = 1.62) after the removal of the PCL, and similarly, the mean medial flexion gap increased from 15.7 mm (SD = 2.36) to 18.8 mm (SD = 1.83). The flexion medial gap increased by an average of 1 mm more than the flexion lateral gap after PCL resection (Table 3).

**Table 3:** Initial robotic gap captures (with and without PCL) from mechanical alignment start-point *Paired student t-test PCL, posterior cruciate ligament; NS, not significant

Parameter	With PCL retained, mean ± SD	With PCL sacrificed, mean ± SD	p-value
Extension - Lateral (mm)	23.5 ± 1.90	23.5 ± 1.88	NS
Extension - Medial (mm)	19.1 ± 2.25	19.3 ± 1.93	NS
Flexion - Lateral (mm)	22.7 ± 1.66	24.5 ± 1.62	0.000*
Flexion - Medial (mm)	15.7 ± 2.36	18.8 ± 1.83	0.000*

Further analysis of the 70 knees shows the true influence of PCL resection on femoral bony resections. All knees, after rebalancing, showed a significant reduction in the medial and lateral posterior femur bone resection thickness. The posteromedial femur cut was reduced from 9.6 mm to 7.1 mm, and the posterolateral femur cut was reduced from 4.8 mm to 2.7 mm. There was a trend toward a reduction of femoral external rotation without the PCL; however, it was not statistically significant. There was no significant change in the mean medial or lateral distal femoral resection (Table 4).

**Table 4:** Impact of PCL resection on balance and bone-cut thickness PCL, posterior cruciate ligament; MA, mechanical alignment; FA, functional alignment; NS, not significant *Paired student t-test

Parameter		Mean (SD) (N = 70)		
		With PCL	Without PCL	p-value
Initial gap captures with MA start (mm)	Extension - Lateral	23.2 ± 2.0	23.2 ± 1.9	NS
	Extension - Medial	19.6 ± 1.8	19.7 ± 1.7	NS
	Flexion - Lateral	22.3 ± 1.6	24.4 ± 1.6	0.000*
	Flexion - Medial	16.5 ± 1.9	19.7 ± 1.6	0.000*
Final gap captures after FA positioning (mm)	Extension - Lateral	18 ± 0	18.1 ± 0.5	NS
	Extension - Medial	17.7 ± 0.4	17.9 ± 0.7	NS
	Flexion - Lateral	18.1 ± 0.3	18.1 ± 0.5	NS
	Flexion - Medial	17.6 ± 0.6	18 ± 0.6	NS
Femur bone cuts (mm)	Postero - Medial	9.6 ± 1.1	7.1 ± 1.4	0.000*
	Postero - Lateral	4.8 ± 1.6	2.7 ± 1.4	0.000*
	Distal - Medial	6.1 ± 1.3	6.5 ± 1.6	NS
	Distal - Lateral	3.5 ± 1.5	3.8 ± 1.7	NS
Component positioning (degrees)	Femur - External rotation	3.7 ± 1.4	3.3 ± 1.4	NS (0.07)
	Femur - Varus	1.13 ± 1	1 ± 1	NS
	Tibia - Varus	2.3 ± 0.9	2.3 ± 1	NS

## Discussion

This study compared the impact of PCL retention and sacrifice on soft tissue balance in patients undergoing primary RA-TKA for varus deformities. The results revealed that a high proportion of knees could be pre-balanced with the PCL retained, using functional alignment with Mako. PCL resection consistently led to a preferential increase in the flexion gap, with minimal change in the extension gap [20]. PCL resection did not result in increased correction of coronal deformities, as reported by Kayani et al. [16]. PCL resection resulted in an increased flexion gap, with a greater increase in the medial flexion gap than the lateral flexion gap. Surgeons may better understand the long-term effects of implant design and polyethylene wear following a TKA procedure, by knowing the effects of PCL resection [6,21,22].

In knees initially pre-balanced (via 3D computed tomography-based component positioning with functional alignment and dynamic gap assessment) with the PCL retained, reassessment of gap balance and balancing after PCL resection resulted in a significant reduction in posterior-femoral bone resection thickness. The posteromedial cut thickness decreased from 9.6 mm (SD = 1.1) to 7.1 mm (SD = 1.4), which was statistically significant compared to the posterolateral cut, which decreased from 4.8 mm (SD = 1.6) to 2.7 mm (SD = 1.4). These findings suggest that PCL-sacrificing RA-TKA enhances the likelihood of being more bone-conserving in the posterior femur, since the femoral component was either posteriorized or not rotated as much to reduce the larger flexion gap. The trend towards reduced external rotation can be explained by the differential increase in the medial flexion gaps compared to the lateral. A marginal decrease in the external rotation of the femur was needed to rebalance the flexion space, though this was not statistically significant.

Park et al. reviewed outcomes in 30 patients with severe varus deformity (>15 degrees) or fixed flexion deformity (FFD) (>20 degrees) and found that PCL resection increased the mean medial and lateral extension gaps by 1.2 mm and 0.3 mm, respectively, while the mean medial and lateral flexion gaps increased by 4.5 mm and 3.4 mm, respectively [11]. However, this study included cases with severe deformities requiring extensive medial soft tissue releases, which may have influenced medial gap assessments.

In line with the present study, Kadoya et al. reported that PCL release did not affect the extension gap but significantly increased the mean medial flexion gap by 4.8 mm and the mean lateral flexion gap by 4.4 mm (p < 0.001) [12]. Mihalko and Krackow employed a motion-tracking device to assess outcomes in 12 cadaveric knee specimens and found that PCL resection resulted in a significant flexion-extension mismatch, increasing the mean flexion gap by 5.26 mm (SD = 1.9) at rest and 6.4 mm (SD = 2.5) under tension [23]. However, it is important to note that cadavers may introduce non-physiological conditions, leading to significant variations compared to our study.

While concerns exist regarding implant design, the use of an ultra-congruent, highly cross-linked polyethylene liner enhances stability without significant wear concerns. The condylar stabilized (CS) design, which combines the ease of use associated with the PS design with the bone preservation advantages of the CR design, may eliminate the need for PCL "titration" in the future [24-26]. The increase in the flexion gap after PCL resection may explain why deep flexion is better in PS-TKA compared to CR-TKA. Flexion-extension mismatch and mediolateral laxity can lead to greater tibiofemoral translation, which may increase the risk of patellofemoral and medial compartment OA in patients with isolated PCL injuries [27]. This study also demonstrates how robotic technology may serve as a valuable research tool for future studies evaluating ligament and other soft tissue functions in TKA.

The limitations of this study include the relatively small sample size of 70 patients, which may affect the generalizability of the results to a broader population. Additionally, as a single-center study conducted with a specific RA-TKA system, the findings may not be directly applicable to different alignment philosophies or surgical techniques. The exclusion of patients with severe deformities, high BMI, or previous knee surgeries further narrows the applicability of these outcomes. Lastly, while the study examined immediate intraoperative measurements, it did not include long-term follow-up data, limiting conclusions on the enduring impact of PCL resection on knee function and implant longevity.

## Conclusions

This study demonstrates that PCL resection consistently leads to a preferential increase in the flexion gap, with minimal impact on the extension gap. The flexion gap increased more significantly in the medial compartment than the lateral compartment, in approximately a 3:2 proportion. Additionally, PCL resection was associated with decreased posterior femoral bone resection thickness and a trend toward reduced femoral component external rotation. Understanding the ramifications of PCL resection can help surgeons anticipate its effects on implant design and the potential for polyethylene wear, thereby improving long-term outcomes following TKA procedures.
